# Exploring causal relationship between the lipids, immune cells, and leiomyosarcoma: A Mendelian randomization and mediation analysis

**DOI:** 10.1097/MD.0000000000040919

**Published:** 2024-12-27

**Authors:** Xuemei Jin, Chaoyang Jiang, Xia Gan, Xinyun Zou, Hua Li, Ling Zhang

**Affiliations:** a College of Medicine, Southwest Jiaotong University, Chengdu, Sichuan, China; b Department of Oncology, The General Hospital of Western Theater Command, Chengdu, China.

**Keywords:** immune cells, leiomyosarcoma, lipids, Mendelian randomization

## Abstract

This study aimed to delineate the causal nexus between lipids and leiomyosarcoma (LMS), with a particular emphasis on delineating the mediating role of immune cells. Employing a 2-sample Mendelian randomization (MR) framework, we scrutinized the potential association of 179 lipid species with LMS across 179 cases and 314,193 controls. The analysis was underpinned by summary-level data derived from genome-wide association studies. The inverse variance weighting method constituted our primary analytical strategy, augmented by supplementary techniques including MR-Egger, simple mode, weighted median, and weighted mode. To ensure the integrity of our MR inferences, we conducted rigorous horizontal multiplicity, heterogeneity, and Bayesian assessments. Furthermore, a nuanced 2-step Mendelian analysis was undertaken to quantify the extent of immune cell-mediated effects of lipids on LMS. Our comprehensive MR evaluation of 179 lipids species unveiled a significant association between genetically inferred triglyceride levels and an elevated risk of LMS (odds ratio = 2.11, 95% confidence interval = 1.38–3.23, *P* < .001), while inversely showing no effect of LMS on triglyceride levels (odds ratio= 0.99, 95% confidence interval = 0.94–1.04, *P* = .83). Additionally, the examination of 731 immune cell phenotypes highlighted CD8+ natural killer T cells as contributing a 6% mediation in the causal pathway from triglycerides to LMS.

## 1. Introduction

Leiomyosarcoma (LMS) is a notably aggressive subtype of soft tissue sarcoma, originating from smooth muscle cells. Predominantly manifesting in the uterus (uterine leiomyosarcoma) and retroperitoneum, often associated with major blood vessels such as the inferior vena cava, LMS can also develop in the extremities and other soft tissues.^[1]^ Accounting for approximately 10% to 20% of all soft tissue sarcomas cases, LMS is distinguished by its poor prognosis, high heterogeneity, and a significant risk of distant metastasis, with 10-year distant metastasis rates varying from 31% to 71%. The overall 5-year survival rate across all stages remains at approximately 65.3%.^[2–4]^ Despite advances in multimodal therapies, considerable clinical challenges persist, underlining the critical need for elucidating the pathogenesis of smooth muscle sarcomas and advancing novel therapeutic strategies.

Emerging evidence underscores the crucial role of plasma lipids as biomarkers of health, with recent studies establishing a link between alterations in lipid metabolism and cancer prognosis.^[3]^ Moreover, the integration of plasma metabolomics and lipoproteomics has shown promise in predicting LMS progression.

The level of plasma liposomes within tumor cells or among immune cells within the tumor microenvironment significantly influences the immune response to tumors, potentially facilitating immune evasion.^[5]^ During oncologic therapy, immune cells are instrumental in modulating the tumor microenvironment, not only suppressing tumor proliferation and enhancing the anti-tumor immune response but also in directly targeting and destroying tumor cells.^[6,7]^ Numerous research efforts have established natural killer T (NKT) cells as pivotal in tumor immunity, with their capacity to either foster anti-tumor effects or contribute to tumor progression, contingent on the specific cytokine profile expressed and the type of cancer involved.^[6]^ Given the genomic complexity inherent to LMS, the potential of immunotherapy as a viable treatment modality is increasingly apparent.^[8]^

However, the extent to which lipid metabolism interplays with the immune system to influence smooth muscle sarcoma progression remains to be fully understood. This study aims to leverage Mendelian randomization (MR) to explore the relationship between lipid-associated genetic variants and LMS outcomes.^[9,10]^ Specifically, we sought to determine the influence of lipids on LMS progression via immune cell mediation, employing single nucleotide polymorphism (SNP) data as instrumental variables for lipid exposure. Through a detailed 2-sample MR analysis, we endeavored to ascertain the causal relationship between lipid profiles and immune cell characteristics in LMS, further elucidating the potential of lipids to modulate LMS progression through immune system interactions.

## 2. Materials and methods

### 2.1. Study design

**Figure 1.**
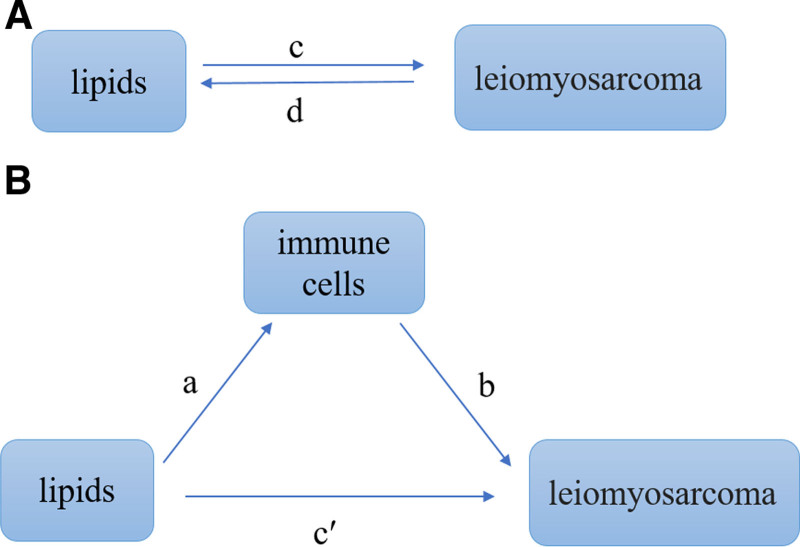
Diagrams illustrating associations examined in this study. (A) The total effect between lipids and LMS. *c* is the total effect using genetically predicted lipids as exposure and LMS as outcome. *d* is the total effect using genetically predicted LMS as exposure and lipids as outcome. (B) The total effect was decomposed into indirect effect using a 2-step approach (where *a* is the total effect of lipids on immune cells, and *b* is the effect of immune cells on LMS) and the product method (*a* × *b*) and direct effect (*c*′ = *c* − *a* × *b*). Proportion mediated was the indirect effect divided by the total effect. LMS = leiomyosarcoma.

This investigation was predicated on the analysis of publicly accessible data sets that have received approval from the institutional review boards of their respective studies. Utilizing a two-sample, 2-way MR approach, our study delved into the causal relationship between lipids and LMS, a subtype of smooth muscle sarcoma. SNPs served as instrumental variables for dissecting the influence of cellular lipidomic features and immune cell markers on the pathogenesis of LMS. Our method involved initial screening for associations between lipids and immune cells with LMS, followed by quantifying the extent to which lipids mediate their effect on LMS via immune cell modulation. The conceptual framework of our study design is illustrated in Figure 1.

### 2.2. Data sources

Our analysis utilized data from LMS encompassing 314,372 individuals, including 179 LMS patients and 314,193 controls, sourced from the Finngen consortium’s genome-wide association (GWAS) summary data (https://www.finngen.fi/en). Additionally, data encompassing 731 immune cell characteristics^[11]^ were procured from the GWAS catalog (https://gwas.mrcieu.ac.uk/), ranging from Ebi-a-GCST0001391 to Ebi-a-GCST0002121. For these data, we utilized the summary statistics from a recent extensive GWAS on blood cell characteristics conducted by the Blood Cell Consortium. This GWAS encompassed 563,085 individuals of European descent. Furthermore, data for 179 lipids from the human plasma liposomes GWAS, derived from the GeneRISK cohort comprising 7174 Finnish individuals, can be accessed in the GWAS catalogue (https://gwas.mrcieu.ac.uk/), under the accession numbers GCST90277238 to GCST90277416. Because it was publicly available aggregated data, no additional ethical approval or consent to participate was required.

### 2.3. Instrumental variable selection and data harmonization

In our selection criteria, we identified SNPs with *P* values below the genome-wide significance threshold (5 × 10^−8^) as IVs to enhance the comprehensiveness of the results and improve sensitivity. To mitigate the impact of related SNPs, all IVs underwent linkage disequilibrium clustering with parameters set to *r*^2^ = 0.001 and a maximum distance of 10,000 kb. Additionally, the *f* statistic formula [*R*^2^(N − 2)/(1 − *R*^2^)]—where *R*^2^ represents the proportion of variance explained by genetic means and N represents the effective sample size of the GWAS—is used to assess the strength of each IV. Only SNPs with an *f* statistic value exceeding 10 were included in the subsequent MR analysis to ensure reliable estimation of genetic variance.^[12–14]^

### 2.4. Statistical analysis

Statistical analyses were conducted in R software version 4.3.2 (https://www.r-project.org), utilizing the TwoSampleMR, VariantAnnotation, and ieugwasr packages for 2-sample MR analyses.^[11,15–27]^ Utilizing methodologies included inverse variance weighting (IVW), MR-Egger, weighted median regression, simple and weighted models, but the IVW method served as the cornerstone for causal estimation, with a significance threshold set at *P* < .05. The statistical power of the MR study was calculated via the mRnd tool (https://cnsgenomics.shinyapps.io/mRnd/). Heterogeneity among SNPs was evaluated using Cochran’s *Q* statistic and funnel plots, while the MR-Egger intercept test and the MR-PRESSO outlier method were applied to identify and adjust for pleiotropic effects. The indirect effects of genetic variants on LMS risk, mediated by immune cells, were quantified using the product of coefficients method, with standard errors determined via the δ method.

## 3. Results

### 3.1. Causal influence of lipids on LMS

A comprehensive 2-sample MR analysis of 179 lipid species identified 15 lipid traits associated with LMS, notably implicating triglycerides (TG) in significantly elevating LMS risk. Utilizing the principal methodology of IVW, the analysis revealed that genetically inferred levels of TG are positively correlated with an increased risk of LMS, indicating a substantial association. See Figure S1, Supplemental Digital Content, http://links.lww.com/MD/O134, which illustrates the identified 15 lipid traits.

### 3.2. Impact of immune cell traits on LMS risk

The IVW model is the primary method for testing causal relationships. Through Mendelian analysis of each Wald ratio for valid SNPs, the most precise effect estimates are derived. With a *P* value threshold of less than 0.05, 33 immune cell traits were identified as having causal relationships with LMS out of 731 features. Notably, a significant positive correlation was observed between CD8+ NKT cells and LMS (IVW: odds ratio [OR] = 1.564, *P* = .005), see Figure S2, Supplemental Digital Content, http://links.lww.com/MD/O134, which depicted identified 33 immune cell traits.

### 3.3. Association between lipids and immune cells

Utilizing methodologies such as IVW, MR-Egger, weighted median regression, simple and weighted models, we further examined the association between TG and CD8+ NKT cells. Data analyzed revealed a positive association between TG and CD8+ NKT cells (IVW: OR = 1.107, *P* = .04), suggesting TG involvement in immune cell modulation. Other analytical methods did not reveal significant causality, emphasizing the importance of the IVW approaches.

### 3.4. Examination of reverse causality between TG and LMS

**Figure 2.**
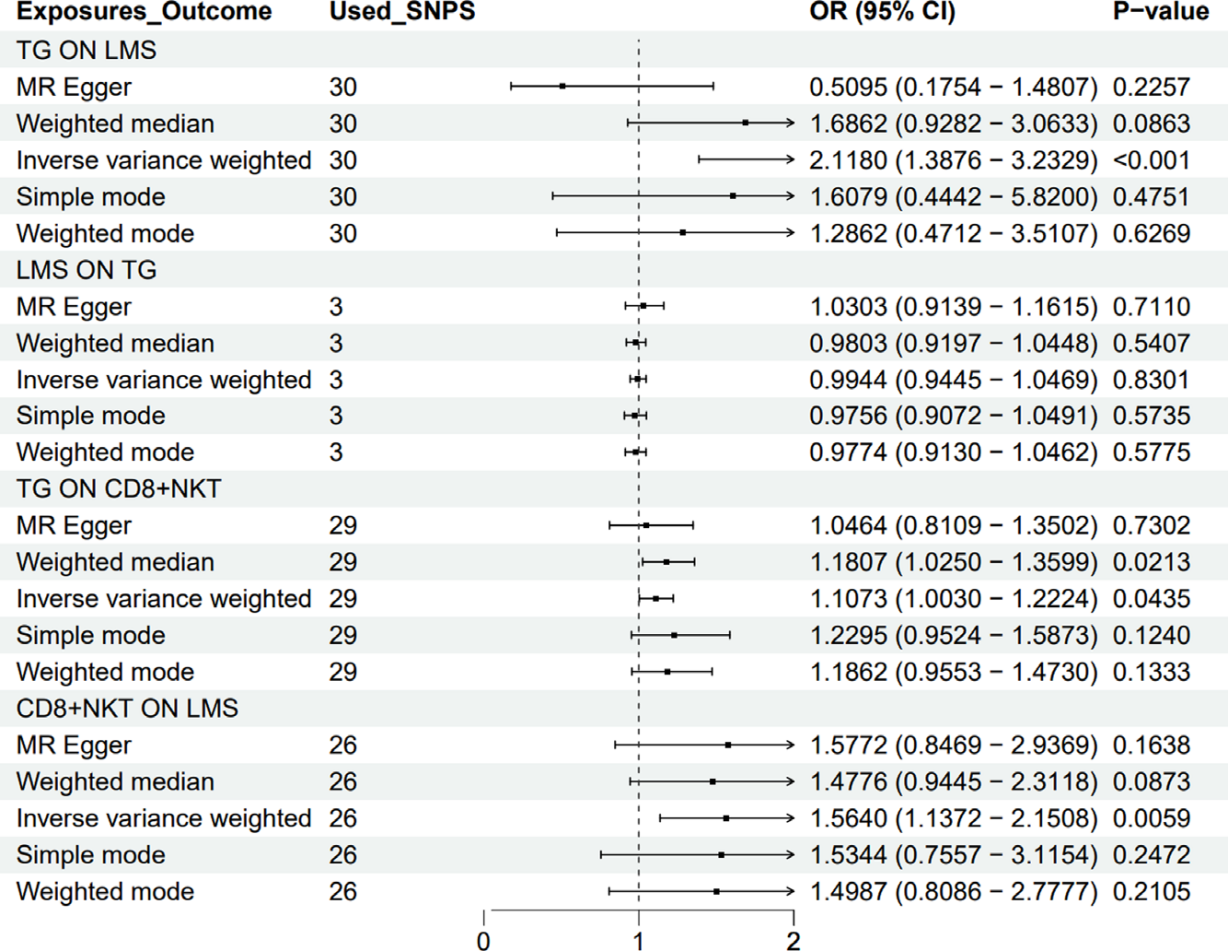
The results are encapsulated through a forest plot, illustrating the causal effects between CD8+ NKT cells, triglycerides, and LMS. CI = confidence interval, LMS = leiomyosarcoma, MR = Mendelian randomization, NKT = natural killer T, OR = odds ratio, SNPs = single nucleotide polymorphisms, TG = triglycerides.

In the exploration of potential reverse causal relationships, employing methods such as IVW, MR-Egger, weighted median regression, simple and weighted models, the analysis revealed that LMS exerts no significant reverse effect on TG levels (IVW: OR = 0.994, *P* = .83), thereby reinforcing the directional causal relationship from TG to LMS. Figure 2 presents the analysis. Table S1, Supplemental Digital Content, http://links.lww.com/MD/O133, presenting detailed SNPs in Figure 2.

### 3.5. CD8+ NKT cells as mediators in the TG-LMS link

**Figure 3.**
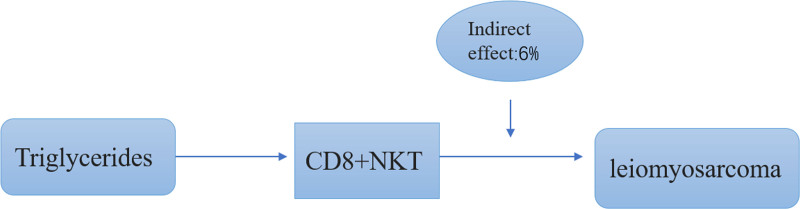
Schematic diagram of the CD8+ T mediation effect. NKT = natural killer T.

In order to determine the causal effect of TG on LMS, we specifically focused on the mediating effect of CD8+ NKT cells on TG. We conducted a mediation analysis to delineate the mediating role of CD8+ NKT cells between TG on LMS. The mediating role of CD8+ NKT cells in the causal pathway from TG to LMS is 6%. The results are shown in Figure 3.

### 3.6. Sensitivity analysis

Sensitivity assessments, including Cochran’s *Q* statistic and the MR-Egger intercept test, alongside MR-PRESSO analysis, indicated no significant heterogeneity or pleiotropy, affirming the robustness of the causal inferences. The stability of causal estimates was further corroborated by a leave-one-out analysis. Refer to Figures S3–S5, Supplemental Digital Content, http://links.lww.com/MD/O134, which demonstrate the sensitivity analysis of the data.

## 4. Discussion

In this study, we utilized publicly available genetic datasets on human plasma liposomes, immune cells, and LMS risk to explore the causal relationships among 179 liposome components and LMS. Recent research has discovered that reprogramming of plasma liposomes impacts tumor response changes, leading to immune escape and inflammatory responses.^[28,29]^ The purpose of this study is to explore the causal relationship between lipids and LMS and to determine if it affects LMS via the mediation of immune cells. Through a 2-sample analysis, the initial phase revealed 15 lipid species significantly associated with LMS. Subsequently, the second phase identified 33 immune traits closely linked to LMS. A comparative analysis indicates that CD8+ NKT cells may serve as a significant regulator in the TG to LMS risk pathway.

Furthermore, our analysis determined the proportion of indirect effects through mediation analysis. The findings indicated that the mediating effect of CD8+ NKT on LMS represents 6% of the total effect. This highlights CD8+ NKT cells as crucial mediators in the link between TG and LMS risk. Previous studies have demonstrated that TG levels are significantly higher in various cancers, including colon, cervical, and non-melanoma skin cancers.^[30]^ Fluctuations in TG levels are implicated in tumor progression, with TG having the ability to activate immune cells and influence their growth and movement.^[31–33]^

Immune regulation is crucial for maintaining immune homeostasis. NKT cells constitute a unique subset of immune cells that participate in various facets of immune responses, including inflammation, tumor immunology, and immune modulation. Research indicates that the CD8-expressing, CD1-independent NKT cell subset is designated as CD8+ NKT cells.^[34]^ CD8+ NKT cells produce a spectrum of cytokines, such as high levels of interferon-γ, and demonstrate potent tumoricidal activity against tumor cells.^[35–37]^ Furthermore, the CD8+ NKT cells suppress the immune response in an antigen-specific manner by effectively killing antigen-bearing DCs.^[38]^ Elevated serum TG levels increase the risk of LMS, which is largely mediated by CD8+ NKT cells. Elevated serum TG levels increase the risk of developing LMS, which is largely mediated by CD8+ NKT cells.CD8+ NKT cells result in an immune escape phenomenon that increases the risk of LMS.

There is no evidence that TG levels indirectly influence LMS progression by affecting CD8+ NKT cells. Our research aims to fill this gap, offering innovative insights into LMS treatment and facilitating the development of novel anti-tumor strategies. In our research, we focused on determining the causal impact of lipids on LMS and elucidating the intermediary role played by immune cells, utilizing an MR framework. Employed within an observational context, this methodology allows our study to approximate the outcomes of a randomized controlled trial, but with the distinct advantages of reduced costs and a lower likelihood of encountering reverse causation.

However, this study is subject to several limitations: The foundation of our evidence is observational research, making it susceptible to confounding factors that may skew the results. The demographic focus on a European cohort lacks cross-validation with other populations, potentially introducing bias. We cannot discount the impact of horizontal pleiotropy and heterogeneity on the study’s findings. Findings indicate that the effect mediated by CD8+ NKT cells on LMS accounts for only 6% of the total effect, underscoring a relatively minor role. This points to the possibility that other mediators exist, suggesting the presence of other mediating pathways worthy of further investigation. In this article, we focus solely on the causal relationships among plasma liposomes, immune cells, and LMS. Further research is necessary to fully understand the mechanisms of their interactions.

## 5. Conclusion

This study delineates the causal interplay between liposomes, immune cells, and LMS risk, identifying a positive association between LMS and the serum levels of triacylglycerol and CD8+ NKT, and affirming the mediating role of CD8+ NKT cells in the TG-LMS risk pathway. Specifically, elevated serum TG levels can heighten the risk of LMS, which was, to a large proportion, mediated by CD8+ NKT cells. The calculated mediation of CD8+ NKT in the causal pathway of TG on LMS was 6% (Fig. 3).

These findings illuminate the intricate connections between lipid metabolism, immune responses, and LMS progression, paving the way for innovative therapeutic strategies tailored to lipid and immune system modulation.

## Author contributions

**Conceptualization:** Xuemei Jin, Ling Zhang.

**Data curation:** Xuemei Jin, Chaoyang Jiang.

**Formal analysis:** Xuemei Jin.

**Methodology:** Xuemei Jin.

**Project administration:** Xuemei Jin.

**Resources:** Xuemei Jin, Xinyun Zou.

**Software:** Xuemei Jin.

**Validation:** Xuemei Jin, Xia Gan, Hua Li.

**Visualization:** Xuemei Jin, Hua Li.

**Writing—original draft:** Xuemei Jin.

**Investigation:** Chaoyang Jiang, Xia Gan, Xinyun Zou.

**Funding acquisition:** Ling Zhang.

**Supervision:** Ling Zhang.

**Writing—review & editing:** Ling Zhang, Hua Li.

## Supplementary Material


