# Supplementary material for: Exploring causal relationship between the lipids, immune cells, and leiomyosarcoma: A Mendelian randomization and mediation analysis
Source: Medicine (Baltimore). 2024 Dec 27;103(52):e40919. doi: 10.1097/MD.0000000000040919 (PMC11688058; doi:10.1097/MD.0000000000040919)
Supplement: Supplementary file 1 [file medi-103-e40919-s001.pdf]

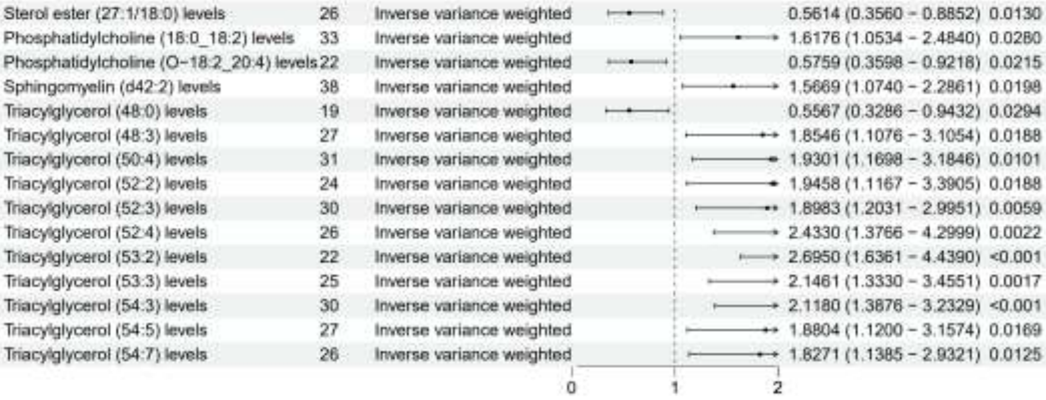

lipid.trait	snp	method	OR (95% CI)	pval
Sterol ester (27:1/18:0) levels	26	Inverse variance weighted	0.5614 (0.3560 − 0.8852)	0.0130
Phosphatidylcholine (18:0_18:2) levels	33	Inverse variance weighted	1.6176 (1.0534 − 2.4840)	0.0280
Phosphatidylcholine (O−18:2_20:4) levels	22	Inverse variance weighted	0.5759 (0.3598 − 0.9218)	0.0215
Sphingomyelin (d42:2) levels	38	Inverse variance weighted	1.5669 (1.0740 − 2.2861)	0.0198
Triacylglycerol (48:0) levels	19	Inverse variance weighted	0.5567 (0.3286 − 0.9432)	0.0294
Triacylglycerol (48:3) levels	27	Inverse variance weighted	1.8546 (1.1076 − 3.1054)	0.0188
Triacylglycerol (50:4) levels	31	Inverse variance weighted	1.9301 (1.1698 − 3.1846)	0.0101
Triacylglycerol (52:2) levels	24	Inverse variance weighted	1.9458 (1.1167 − 3.3905)	0.0188
Triacylglycerol (52:3) levels	30	Inverse variance weighted	1.8983 (1.2031 − 2.9951)	0.0059
Triacylglycerol (52:4) levels	26	Inverse variance weighted	2.4330 (1.3766 − 4.2999)	0.0022
Triacylglycerol (53:2) levels	22	Inverse variance weighted	2.6950 (1.6361 − 4.4390)	<0.001
Triacylglycerol (53:3) levels	25	Inverse variance weighted	2.1461 (1.3330 − 3.4551)	0.0017
Triacylglycerol (54:3) levels	30	Inverse variance weighted	2.1180 (1.3876 − 3.2329)	<0.001
Triacylglycerol (54:5) levels	27	Inverse variance weighted	1.8804 (1.1200 − 3.1574)	0.0169
Triacylglycerol (54:7) levels	26	Inverse variance weighted	1.8271 (1.1385 − 2.9321)	0.0125

Figure S1 MR analysis shows 15 lipid traits correlated with LMS

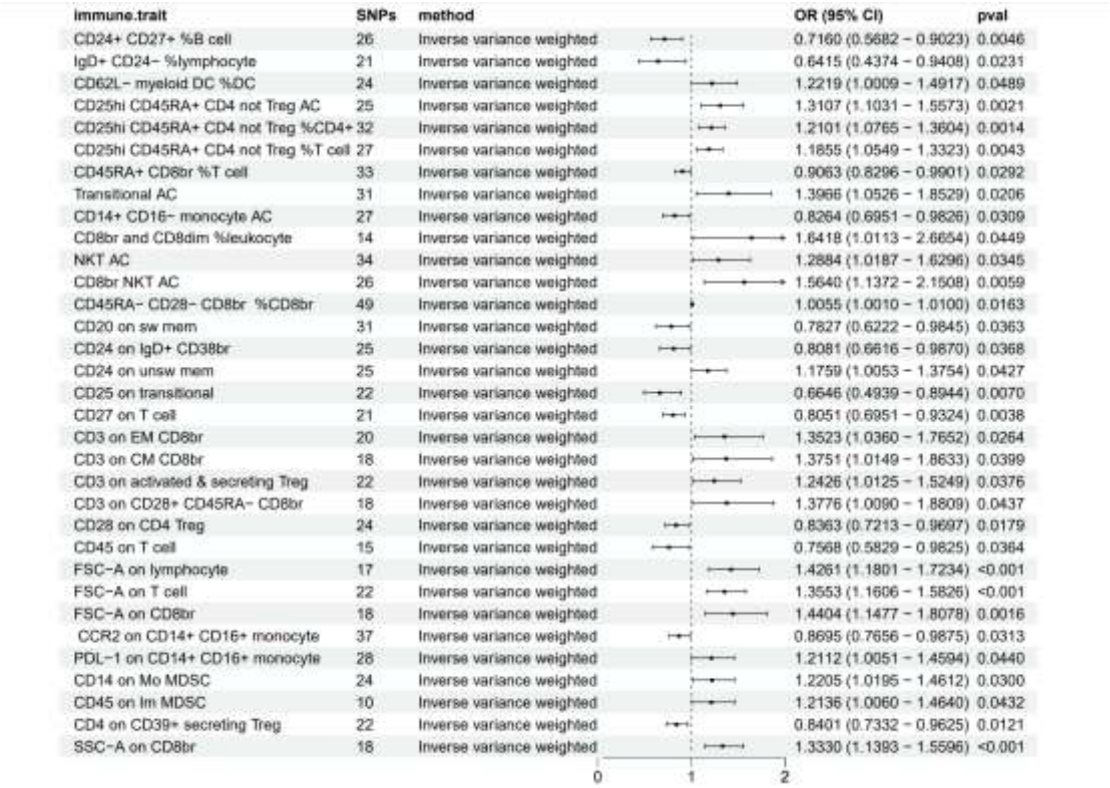

Figure S2 MR analysis shows 33 immune cell traits correlated with LMS

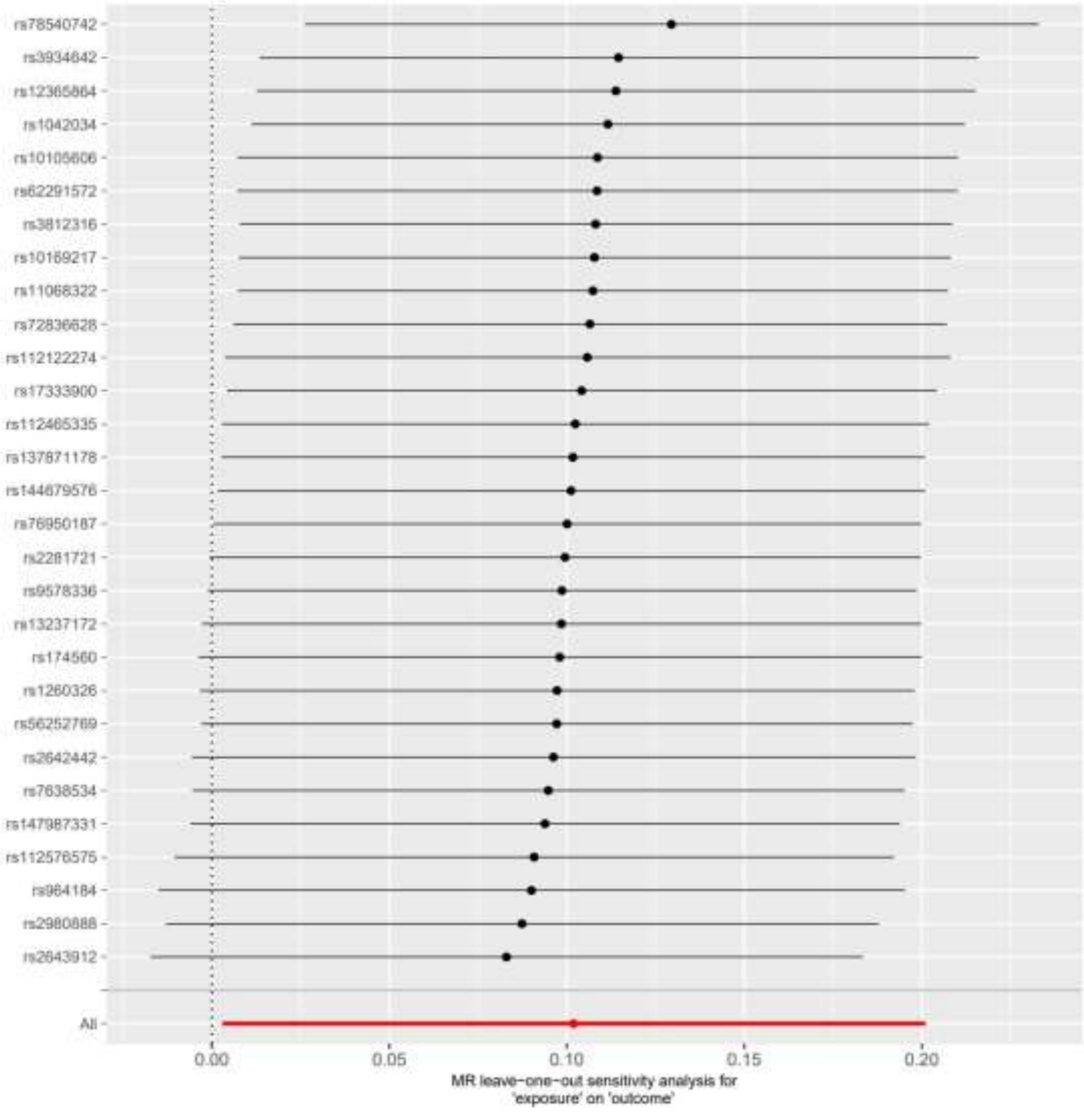

FigureS3 Sensitivity analysis of TG on CD8+NKT

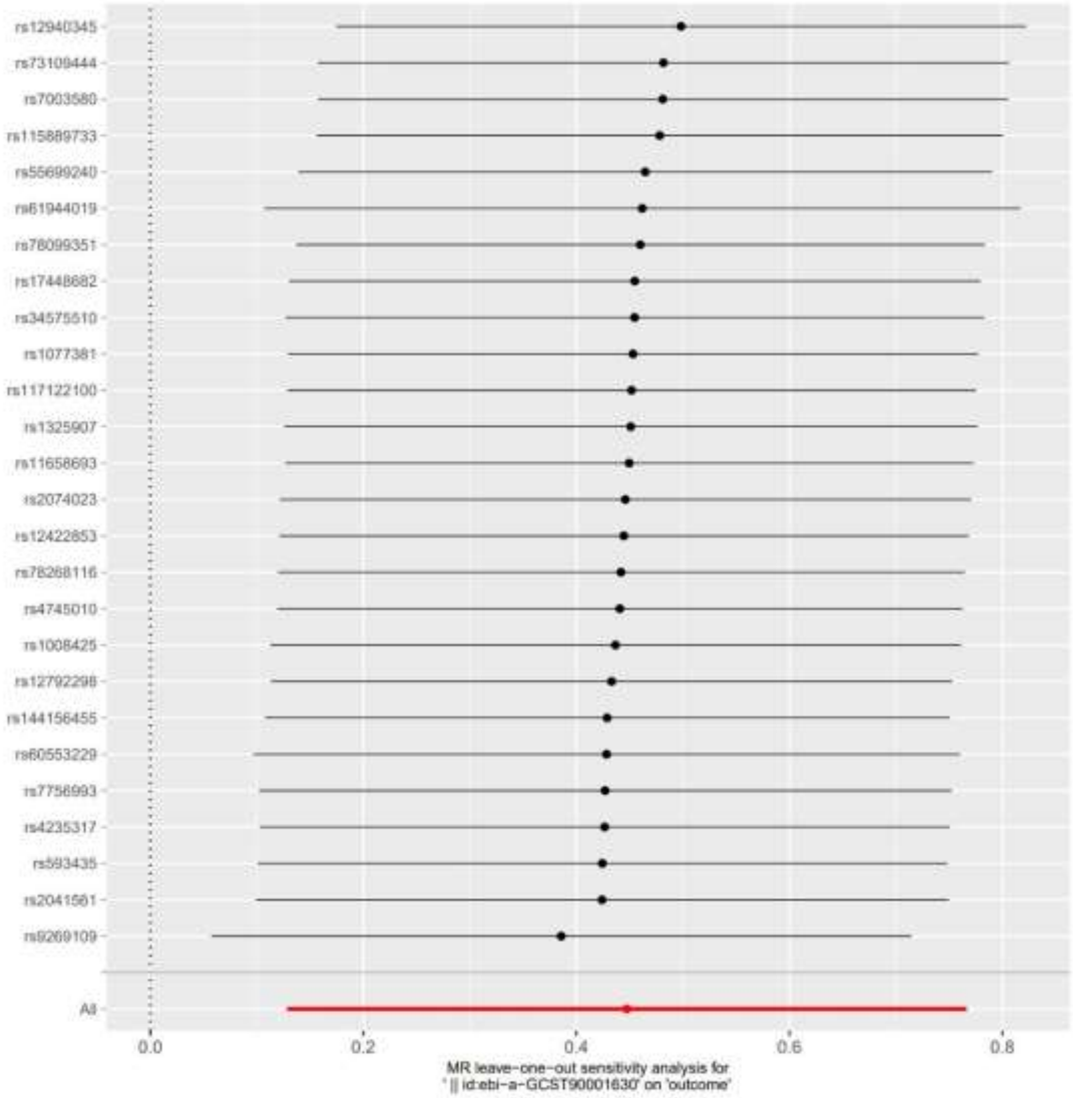

Figure S4 Sensitivity analysis of CD8+NKT on LMS

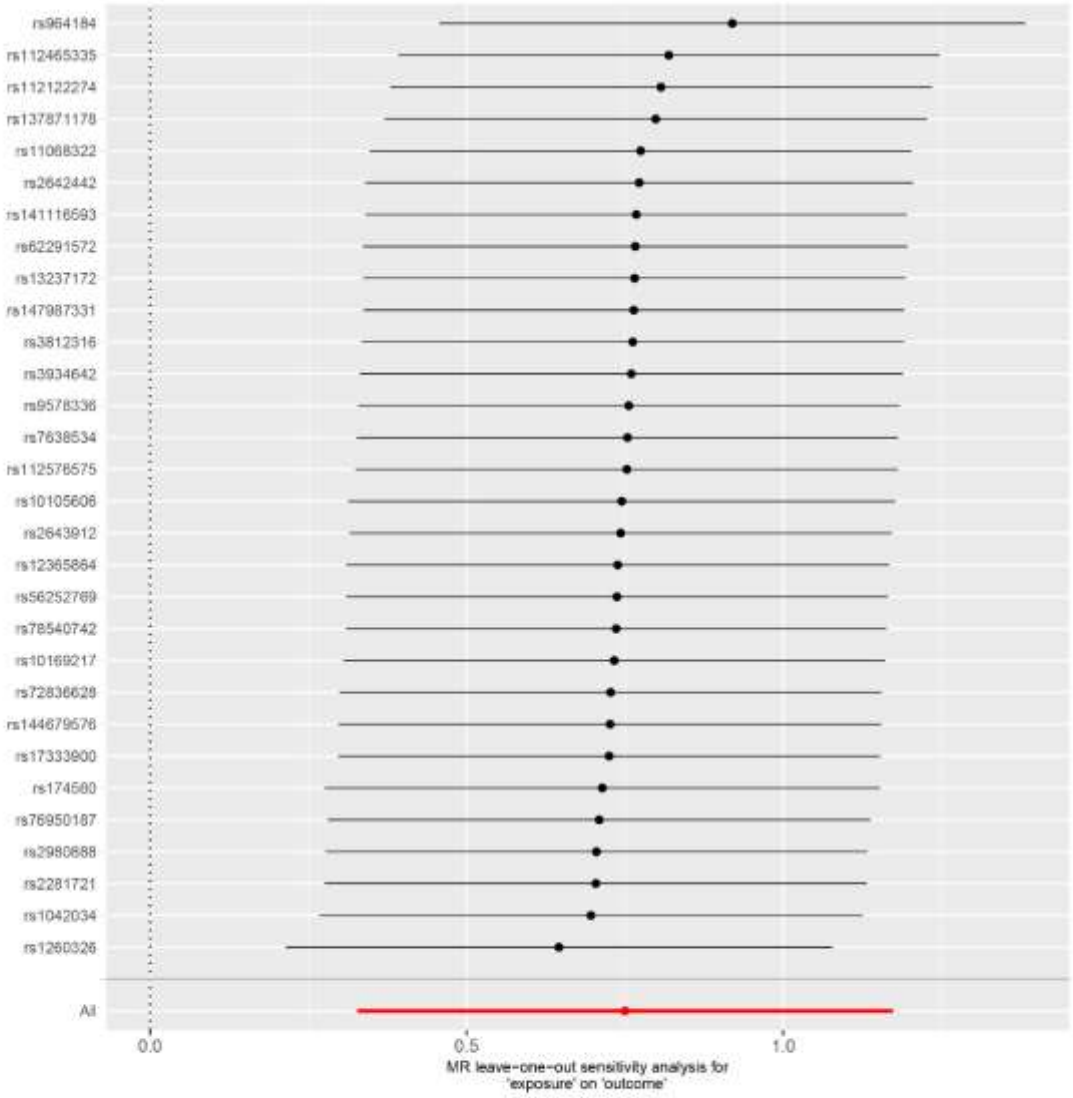

Figure S5 Sensitivity analysis of TG on LMS
